# Effect of exercise on memory function and daily activities after colorectal cancer surgery: an exploratory secondary analysis from the randomised trial PHYSSURG-C

**DOI:** 10.1136/bmjopen-2026-120096

**Published:** 2026-09-23

**Authors:** Carolina Ehrencrona, Ying Li, Kevin Afshari, Eva Angenete, Sandra Buratti, Charlotta Larsson, Aron Onerup, Anette Wedin, Eva Haglind

**Affiliations:** 1 Department of Surgery, SSORG - Scandinavian Surgical Outcomes Research Group, Institute of Clinical Sciences, Sahlgrenska Academy, University of Gothenburg, Gothenburg, Sweden; 2 Biostatistics, School of Public Health and Community Medicine, Institute of Medicine, Sahlgrenska Academy, University of Gothenburg, Gothenburg, Sweden; 3 Department of Surgery, Sahlgrenska University Hospital, Gothenburg, Region Västra Götaland, Sweden; 4 Department of Psychology, University of Gothenburg, Gothenburg, Sweden; 5 Department of Pediatric Oncology, Sahlgrenska University Hospital, Gothenburg, Region Västra Götaland, Sweden; 6 Department of Pediatrics, Institute of Clinical Sciences, Sahlgrenska Academy, University of Gothenburg, Gothenburg, Sweden

**Keywords:** Colorectal surgery, Cognition, Exercise, Cancer

## Abstract

**Objectives:**

To explore the effect of a short-term unsupervised exercise intervention on memory function and activities of daily living after surgery for colorectal cancer.

**Design:**

Secondary analysis within a randomised controlled open label trial with central computerised 1:1 parallel allocation.

**Setting:**

One university and five regional hospitals in Sweden. Recruitment period 2015–2020.

**Intervention:**

Moderately intense aerobic physical activity 30 minutes a day, 2 weeks before and 4 weeks after surgery.

**Participants:**

Patients with colorectal cancer undergoing elective curative surgery. Of the 761 randomised, 631 completed the study and were analysed (intervention 296/379, control 335/382). Median age 70 years, 61% men.

**Outcome measures:**

Self-reported memory function and activities of daily living. Questionnaires were distributed at inclusion and at 4 weeks and 12 months postoperatively.

**Results:**

There was no difference in self-reported memory function between the groups at 4 weeks. At 12 months, participants in the intervention group were less likely to report being told they had poor memory (OR 0.61, 95% CI 0.38 to 0.98). Regarding daily activities, participants in the intervention group were more likely to report a greater ability to shop for food or other necessities at 4 weeks (OR 1.53, 95% CI 1.07 to 2.20). No other significant differences were seen.

**Conclusion:**

This exploratory study with secondary data, nested in a multicentre randomised trial, found no substantial effects of a short-term unsupervised exercise intervention on memory function or the ability to perform daily activities in patients with colorectal cancer. An exercise intervention of this duration and intensity could be insufficient for improvement compared with standard care.

**Trial registration number:**

NCT02299596.

**STRENGTHS AND LIMITATIONS OF THIS STUDY**

- A large multicentre randomised controlled trial with few exclusion criteria enhances generalisability.
- Questionnaire response rates were 80% or higher throughout follow-up.
- The sample size was not calculated for secondary outcomes.
- No objective measures or established scoring instruments were used, limiting comparability.

## Introduction

 Cognitive function can decline in patients after colorectal cancer treatment.^1 2^ The aetiology of cancer-related cognitive difficulties is multifactorial; cancer itself, but also oncological treatment, as well as surgery, can cause cognitive decline.^3^ Although postoperative cognitive decline is often temporary, some patients, especially the elderly, may experience persistent symptoms.^4^

Subjective complaints from patients with cancer on decline in cognitive function and the impact on daily life usually concern memory and executive function.^5^ A decline in executive function can require increased efforts to resume activities after interruption, plan ahead, make decisions and follow conversations.^6^ Both memory and executive function are linked to the ability to perform daily activities, especially instrumental activities of daily living, such as household chores and shopping.^7^ A reduced ability to perform daily activities can be a sign of cognitive decline.^8^

Habitual physical activity has a modest protective effect on cognitive decline,^9^ and physical exercise has been suggested as a possible intervention to mitigate cancer-related cognitive impairment^5 10^ and postoperative cognitive decline.^11^ However, recent reviews have found that evidence regarding the effects of physical activity on cancer-related cognitive difficulties is inconclusive for both short and long-term effects.^12 13^ Studies of long-term effects were underrepresented, and most assessments of cognitive function occurred at the end of intervention.^12^ Colorectal cancer is the third most common cancer worldwide.^14^ Yet to date, few studies on the effect of physical activity on cognition after cancer treatment have included cohorts of patients with colorectal cancer, and among these, randomised trials were small or focused on effects after neoadjuvant or adjuvant treatment.^12 13^

PHYSSURG-C was a large multicentre randomised controlled trial of an unsupervised, short-term physical activity intervention in patients scheduled for elective surgery for colorectal cancer. The trial was primarily designed to investigate recovery after surgery. This exploratory study used data from PHYSSURG-C with the aim of exploring the effect of the physical activity intervention, compared with usual care, on self-reported memory function and the ability to perform daily activities at 4 weeks and 12 months after surgery.

## Method

PHYSSURG-C was an open-label randomised controlled superiority trial which recruited patients from five regional and one university hospital from 2015 to 2020. The intervention involved: (1) preoperative inspiratory muscle training during approximately 2 weeks before surgery and (2) increased physical activity beginning about 2 weeks before surgery and continuing for 4 weeks after discharge. The trial was registered at ClinicalTrials.gov (NCT02299596) on 17 November 2014, and the protocol has been published.^15^ Results for the primary endpoints, at 4 weeks and 12 months after surgery, have been published.^16 17^

### Patient and public involvement

Patients and the public were not involved in the design, conduct or reporting of this trial.

### Participants

Patients aged 20 years or older with colorectal cancer and scheduled to undergo elective surgery with curative intent were eligible for inclusion. Exclusion criteria were emergency surgery, local excision, cytoreductive surgery with hyperthermic intraperitoneal chemotherapy or inability to perform the intervention or to understand instructions and questionnaires (ie, language barrier). Patients were included at the time of diagnosis by hospital staff at the Departments of Surgery in participating hospitals.

### Randomisation and intervention

Randomisation was centralised and computerised with a 1:1 allocation and a block size of 4 and parallel groups. Stratification was made by surgical technique (laparoscopic or open) and by tumour site and neoadjuvant radiotherapy (colon, rectum without radiotherapy or rectum with radiotherapy). Neither participants nor care providers were blinded.

All participants received general information about the aim of the study and followed local routines for pre- and postoperative care. Participants in the intervention group met individually with a physiotherapist to discuss suitable activities to achieve a moderately intense level of exercise. They were instructed to perform inspiratory muscle training and to add 30 min of aerobic activity of their own choice daily to their normal routine 14 (±4) days before surgery. They were also instructed to resume the same level of physical activity as the preoperative phase after discharge and maintain this level for 4 weeks. All participants recorded the nature of the performed exercise, duration and type of activity in a diary. All participants received a phone call from a research nurse to enquire about any adverse events, to provide reminders to increase resistance during inspiratory muscle training and to motivate participation. The procedure has previously been described in detail.^16^

### Data collection

Baseline data were collected by electronic and paper case report forms. Data regarding tumour classification and the surgical procedure were retrieved from the Swedish ColoRectal Cancer Registry. Data on complications, neoadjuvant and adjuvant treatments were extracted from medical charts.

Participants received questionnaires preoperatively at inclusion and at 4 weeks and 12 months after surgery. The questionnaires and exercise diaries were returned to the central study secretariat.

### Outcomes

Outcome data were obtained from questionnaires administered at the 4-week and 12-month follow-ups in the original trial. All questions and response categories can be found in online supplemental file 1), (online supplemental table S1). These questions were originally developed by the research group to be used in cohort studies on quality of life in patients with cancer.^18–20^ The questions were constructed after content analysis of semi-structured patient interviews, and then underwent expert and patient face validation (see Reinwalds *et al*^19^ for a more detailed description of the procedure). The questions on memory function and daily activities were framed to cover the duration of the past month.

The memory function outcome was measured with one general question regarding whether anyone had told or suggested to the participant that they had *poor memory* and two questions on subjective memory difficulties (*recalling factual information* and *remembering names*). The questions had the same seven response categories based on frequency, ranging from never to all the time. In the analysis, three of the categories corresponding to daily frequency were combined into one, representing *daily occurrence*.

The daily activities outcome was measured with one question on the proportion of daily activities performed, with five response categories ranging from none to all, and three questions on the ability to perform specific activities (*household tasks, shopping* and *socialising)*, with four response categories ranging from no to great ability. Those selecting the response category *not applicable* for these questions were excluded from the analyses.

### Statistical analysis

A power analysis was performed for the primary outcome of the PHYSSURG-C trial (ie, physical recovery) in a preplanned interim analysis. No posterior power analysis has been performed for the outcomes examined in this study (ie, memory function and daily activities). Statistical analyses were performed according to a predefined statistical analysis plan (online supplemental file 2) for these outcomes.

This study evaluated secondary outcomes of the PHYSSURG-C trial, and these analyses were regarded as exploratory without the intention of making confirmatory claims. Therefore, no correction for multiple testing was applied.

The primary analysis included the intention-to-treat population, as randomised. Proportional odds logistic regression models were fitted to estimate the effect of treatment (intervention vs control) on ordinal outcomes assessed at 4 weeks and 12 months, including memory function and daily activities. The proportional odds model was used to maintain multiple response levels and their order within the model. The proportional odds assumption was evaluated using the Brant test and held across models. Analyses were adjusted for stratification factors used in the trial (surgical method, tumour location and neoadjuvant therapy) and preoperative values of the corresponding outcome variables. Missing data were handled using listwise deletion; no imputation was performed. Results are presented using OR for the intervention vs the control with 95% CI and p value.

A per-protocol analysis was performed on the participants in the intervention group who reported aerobic exercise or inspiratory muscle training at least 8 days, between inclusion and surgery, that is, no less than 80% of the minimum (ie, 14−4) available days. The remaining participants in the intervention arm were excluded while all participants in the control arm were included in the analysis.

An intervention with pre- and postoperative physical activity is likely to have the largest effect in patients who have a high risk of prolonged recovery and who are habitually relatively physically inactive.^21 22^ Therefore, a supporting per-protocol subgroup analysis was performed for high-risk inactive patients. The subgroup was defined by fulfilling the two following conditions: (1) age >70 years or ASA classification ≥3 or long neoadjuvant therapy and (2) low physical activity, defined as ≤2 on the Saltin-Grimby physical activity level scale.

Further, a post hoc time-effect analysis with an unadjusted proportional odds logistic regression model was carried out for descriptive purposes. This was done to examine if there were any detectable changes in the outcomes over time as we reasoned that if there were no changes it would be unlikely that the intervention could have shown any effect. As this was a post hoc analysis intended to evaluate the measurement, and not compare groups, the whole cohort was pooled in the analysis. Compared timepoints were preoperatively to 4 weeks, preoperatively to 12 months and 4 weeks to 12 months.

All statistical analyses were performed. in R, V.4.3.2.

## Results

Of the 761 randomised participants, a total of 631 completed the study: 296 in the intervention group and 335 in the control group (figure 1). The lowest return rate for the questionnaire was 80% at the 12-month follow-up in the control group. The high-risk subgroup included 252 participants, 93 of whom were in the intervention group. In the intervention group, 197 participants complied with the intervention as per-protocol (ie, performed at least eight preoperative days of exercise). The study population had a median age of 70 years, and 39% were women. The groups were balanced regarding demographic variables (table 1).

**Figure 1:**
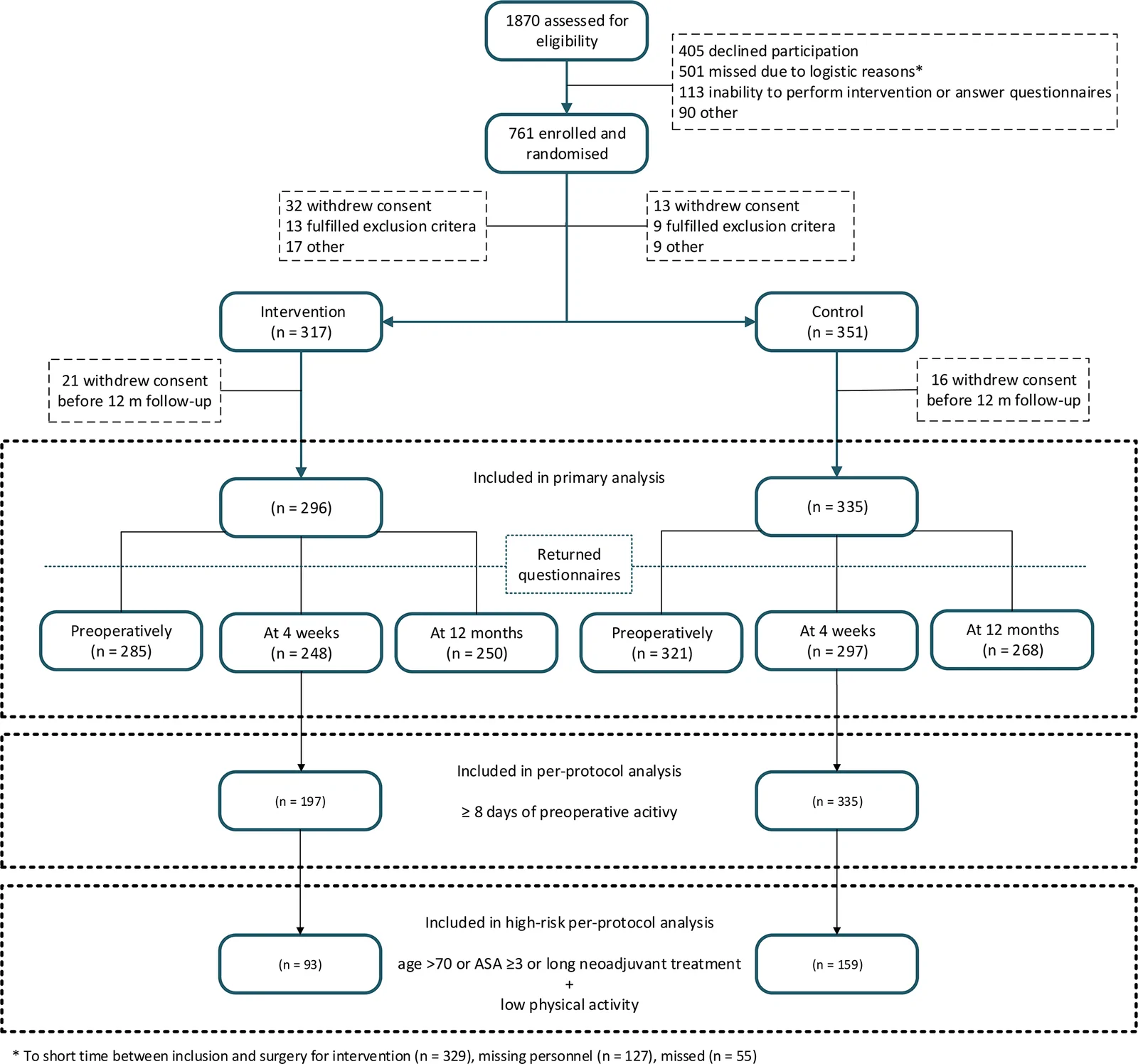
. Flow chart showing participant progression from assessment for eligibility to study completion. The number of returned questionnaires at each timepoint is displayed as well as the per-protocol and high-risk subgroup populations. ASA - American Society of Anesthesiologists classification

**Table 1:** Demographics

	Control n=335	Intervention n=296
Age*	70 (62, 77)	70 (62, 76)
Female/male	132 (39%)/203 (61%)	116 (39%)/180 (61%)
BMI*	25.3 (23.1, 27.8)	25.5 (23.0, 28.5)
Missing	3	0
Born in Sweden	273 (87%)	243 (86%)
Missing	20	12
Education level		
Compulsory school	69 (22%)	54 (19%)
Upper secondary	139 (44%)	120 (43%)
University	107 (34%)	105 (38%)
Missing	20	17
Marital status		
Married or cohabiting	240 (76%)	205 (72%)
Single or living alone	76 (24%)	78 (28%)
Missing	19	13
Current regular smoker	8 (2.5%)	8 (2.8%)
Missing	19	14
ASA classification		
1	53 (18%)	50 (19%)
2	177 (61%)	159 (61%)
3	62 (21%)	47 (18%)
4	0 (0%)	3 (1.2%)
Missing	43	37
Tumour site		
Colon	165 (49%)	151 (51%)
Rectum	170 (51%)	145 (49%)
Neoadjuvant therapy		
No radio- or chemotherapy	215 (64%)	191 (65%)
Long chemo(radio)therapy	39 (12%)	35 (12%)
Short-term radiotherapy	81 (24%)	70 (24%)
Surgical technique		
Laparoscopic	201 (64%)	188 (68%)
Open	111 (36%)	88 (32%)
Missing	23	20
pTNM-stage		
0	1 (0.3%)	1 (0.4%)
I	86 (28%)	78 (29%)
II	91 (30%)	66 (24%)
III	103 (34%)	109 (40%)
IV	26 (8.5%)	16 (5.9%)
Missing	28	26
Postoperative confusion	16 (4.8%)	6 (2.0%)
Received adjuvant treatment	115 (34%)	113 (38%)

* Median (Q1, Q3).

ASA, American Society of Anesthesiologists; BMI, Body Mass Index; pTNM, Pathological Tumor Node Metastatis.

### Memory function

Frequency tables for all questions on memory function can be found in online supplemental file 1 (online supplemental table S2a). The results of the primary adjusted analyses are presented in figure 2 and online supplemental file 1, (online supplemental table S3). There were no differences between the intervention and control groups at 4 weeks regarding any of the outcomes. At 12 months, a difference between the groups was observed regarding whether the participant was told or suggested they had poor memory (OR 0.61, 95% CI 0.38 to 0.98) favouring the intervention group.

**Figure 2:**
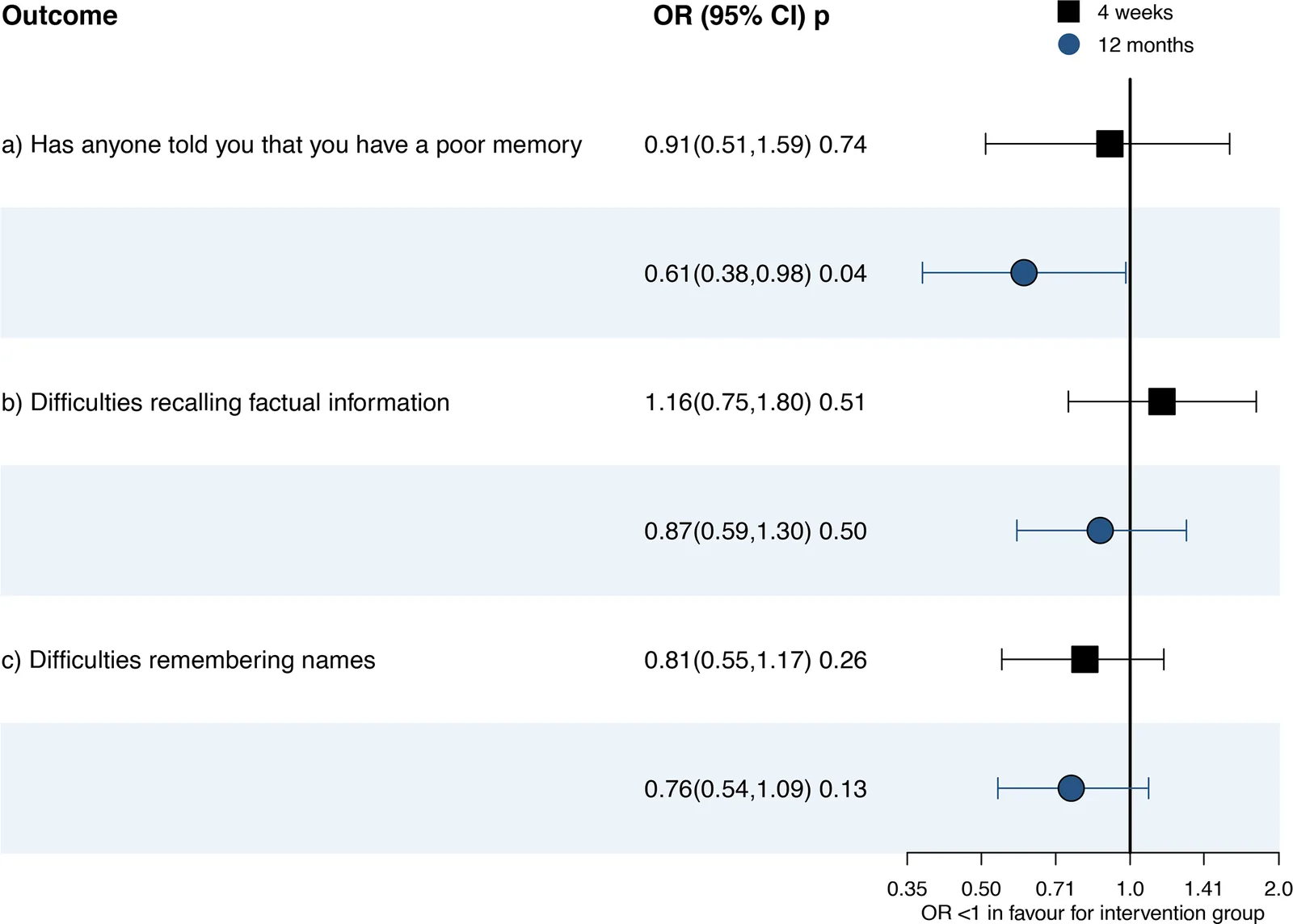
. Forest plot displaying results from adjusted proportional odds logistic regression models of memory function outcomes at 4 weeks (square) and 12 months (circle). The models include response categories: no; yes, <1 time/week; yes, 1 time/week; yes, 3 times/week; yes, daily (**a–c**). The results are presented with odds ratios (OR), 95% CIs and p values (p). An OR <1 indicates a higher likelihood that the intervention group reported memory difficulties occurring less often than the control group.

The results of the analyses for the high-risk subgroup and the per-protocol population showed no significant effects (online supplemental file 1), (online supplemental table S4–S5). The time-effect analyses for the whole cohort did not show increased memory difficulties at 4 weeks compared with preoperative levels. In contrast, difficulties remembering names were less pronounced at 4 weeks compared with preoperative levels (OR 0.67, 95% CI 0.51 to 0.87). At 12 months, there was a decline in memory function compared with levels both preoperatively and at 4 weeks for all questions (online supplemental file 1), (online supplemental table S6).

### Daily activities

Frequency tables for all questions on daily activities are in online supplemental file 1 (online supplemental table S2b). All primary adjusted analyses are presented in figure 3 and online supplemental file 1 (online supplemental table S3). The proportion of daily activities performed did not differ between the groups at 4 weeks (OR 0.96, 95% CI 0.69 to 1.33). Regarding specific tasks, the intervention group had a higher ability to shop for food or other necessities at 4 weeks (OR 1.53, 95% CI 1.07 to 2.20). There was no statistically significant difference between the groups at 12 months.

**Figure 3:**
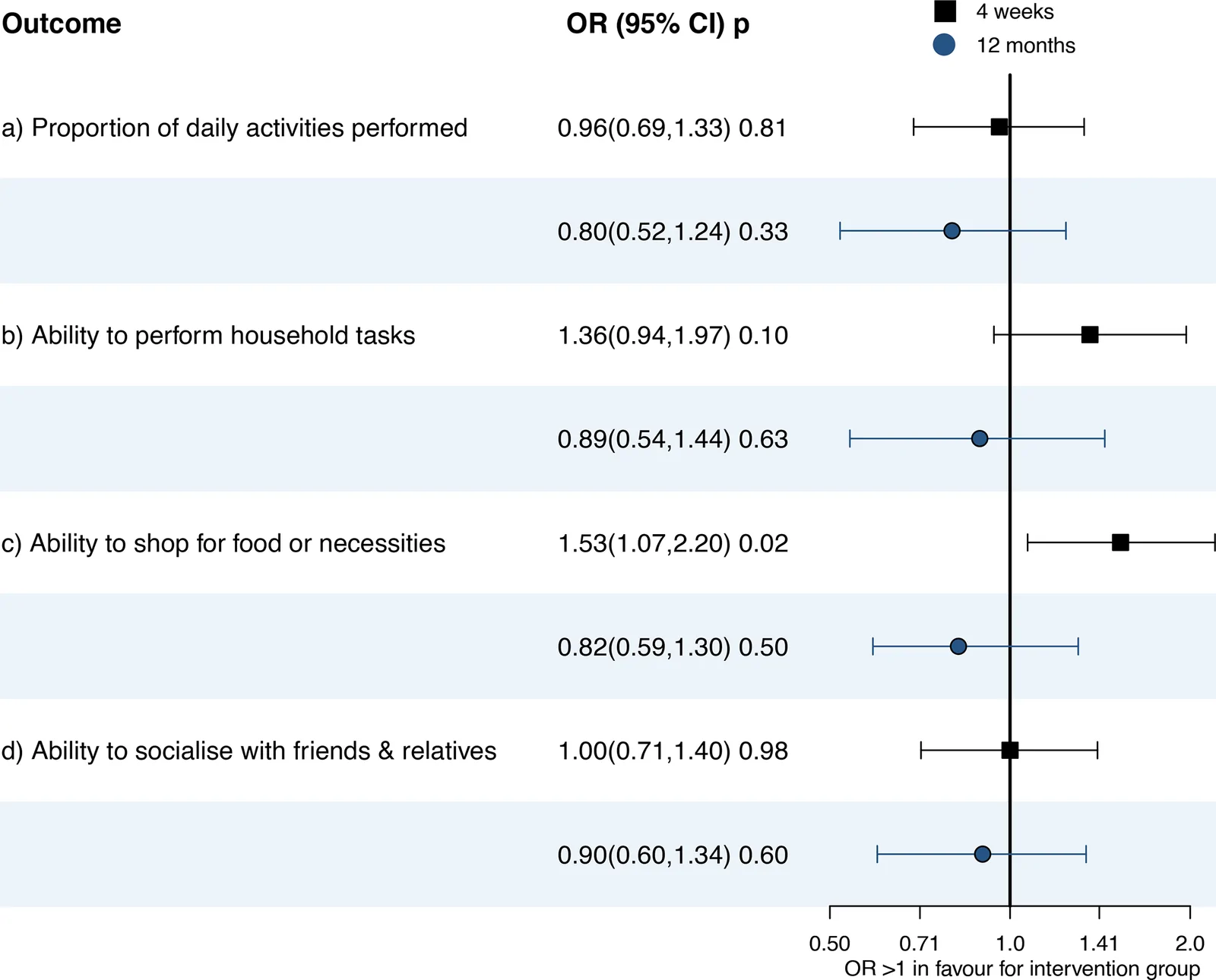
. Forest plot displaying results of adjusted proportional odds logistic regression models of daily activities outcomes at 4 weeks (square) and 12 months (circle). The models include response categories: none; less than half; about half; more than half (**a**), and no ability; little ability; moderate ability; great ability (**b–d**). The results are presented with odds ratios (OR), 95% CIs and p values (p). An OR >1 indicates a higher likelihood that the intervention group reported having a greater ability than the control group.

The per-protocol analysis showed the same pattern as the primary analysis, with a difference at 4 weeks in the ability to shop for food or other necessities (online supplemental file 1), (online supplemental table S4). There was no difference between the groups in the high-risk subgroup analysis (online supplemental file 1), (online supplemental table S5). The time-effect analysis for the whole cohort showed that the ability to perform daily activities had declined 4 weeks after surgery compared with preoperatively and then recovered from 4 weeks to 12 months (online supplemental file 1), (online supplemental table S6).

## Discussion

In our exploratory study, nested in a multicentre randomised controlled trial, we found no clear overall pattern in the results. This unsupervised, short-term, moderately intense aerobic pre- and postoperative exercise intervention had limited effects on memory function and the ability to perform daily activities based on self-reported outcomes.

This study contributes to the knowledge base on physical activity interventions to mitigate cancer-related cognitive decline through the analysis of data from a large randomised controlled trial with long-term follow-up and a population of patients with colorectal cancer. Previous evidence is primarily based on breast cancer cohorts and randomised trials with smaller sample sizes.^12 13^ Brunet and Sharma^12^ call for better diversity and representation in studies exploring physical activity and cognition among patients with cancer, both in terms of cancer type and participant characteristics. A major strength of this trial was the small number of exclusion criteria, increasing external validity. Screened but not randomised patients were comparable in age and sex to those included and randomised.^16^ The compliance rate with the intervention was comparable with other studies of exercise prehabilitation among colorectal cancer surgery patients,^23^ and the response rate for questionnaires was high with over 80% at all timepoints.

A limitation of this study is that the PHYSSURG-C trial was not designed to evaluate the intervention on cognitive outcomes. The sample size was not calculated for secondary outcomes and thus the study could be underpowered for these exploratory analyses. Objective measurements, such as neurocognitive testing, are more sensitive and could have detected nuanced effects on memory function better than subjective measurements.^24^ Also, the outcomes are based on questions developed by our group that have not been validated against other instruments or measurements. A further limitation was that the analysis concerned one direct cognitive function (ie, memory), and the ability to perform daily activities provides only an indirect indication of cognitive functioning abilities. Thus, these results should not be generalised to all cognitive functions.

Of the explored outcomes only the ability to shop for food or other necessities showed a significant difference at 4 weeks both in the primary intention-to-treat analysis and the per-protocol analysis. While grocery shopping is a complex daily activity that demands cognitive abilities, it also requires physical exertion.^25^ The observed effect could be due to increased physical performance in the intervention group or that the exercise intervention encourages participants to spend more time outside of the home. One memory outcome reached statistical significance in the primary analysis, this at 12 months. The estimated effects were in the same direction across all the memory outcomes at 12 months, although with wide CIs. However, the exploratory nature of the analyses and multiple testing without correction, as well as the distribution of ORs, warrants caution regarding the interpretation of the isolated significant p values observed. We did hypothesise that the intervention could be most effective in inactive individuals at a high risk of prolonged recovery. However, this was not found in the subgroup analysis. Notably, the loss of power due to the smaller sample size would have required a substantial effect to be detected in this subgroup compared with the whole study population.

The time-effect analysis showed that memory function had not declined at 4 weeks after surgery compared with preoperative values but then declined at 12 months compared both to levels at 4 weeks and preoperatively. The ability to perform daily activities declined at 4 weeks compared with preoperative values and subsequently improved at 12 months. The literature is inconsistent on the trajectory of decline and improvements in cognitive function after colorectal cancer surgery compared with preoperative values.^2^ The ability to perform daily activities found in our study is consistent with how patients have described their experiences of daily life and recovery trajectory after colorectal cancer surgery.^26^

The primary results from the PHYSSURG-C trial showed no effect on self-reported physical recovery, nor on complications or readmission.^16 17^ Of the secondary outcomes explored, the only effect found was that the intervention group reported less fatigue.^27^ While fatigue has both mental and physical components and can interfere with daily life,^28^ the effect found in this trial was not reflected in the patients’ memory function or the ability to carry out activities in daily life. The effect of exercise interventions on cancer-related cognitive problems is overall inconclusive, and the type of intervention, cancer and treatment modality all presumably exert an impact.^12^ While a meta-analysis showed a summary effect size of 0.27 favouring physical activity interventions, the included study populations consisted completely or predominantly of patients with breast cancer.^13^ As for the duration and intensity of short-term physical activity prehabilitation before abdominal cancer surgery, there is insufficient evidence to support a recommendation.^29^ Better understanding is needed on what type of physical activities, duration, timing, and intensity can support postoperative recovery, including gains in cognitive functions, before implementation in clinical settings. It is possible that the implementation of physical activity interventions after diagnosis may be too late, and rather, efforts should be made to increase physical activity on a population level.

In conclusion, this intervention—unsupervised, short-term moderately intense aerobic physical activity, before and after surgery—had no substantial effect on self-reported memory function or the ability to perform daily activities after colorectal cancer surgery. It is possible that a physical activity intervention of this duration and intensity is insufficient to substantially improve postoperative recovery in patients with colorectal cancer compared with standard care.

## Supplementary material

10.1136/bmjopen-2026-120096 online supplemental file 1

10.1136/bmjopen-2026-120096 online supplemental file 2

## Data Availability

Data are available upon reasonable request.
