# Supplementary material for: Effect of exercise on memory function and daily activities after colorectal cancer surgery: an exploratory secondary analysis from the randomised trial PHYSSURG-C
Source: BMJ Open. 2026 Sep 23;16(9):e120096. doi: 10.1136/bmjopen-2026-120096 (PMC13630044; doi:10.1136/bmjopen-2026-120096)
Supplement: Supplementary file 1 [file bmjopen-16-9-s001.pdf]

SUPPLEMENT

Table S1: List of questions on memory and daily activities and response categories 2
Table S2a: Frequency of responses to the questions on memory function 3
Table S2b: Frequency of responses to the questions on daily activities 4
Table S3: Results of the primary intention-to-treat analysis 5
Table S4: Results of the per-protocol analysis 6
Table S5: Results of the high-risk subgroup per-protocol analysis 7
Table S6: Unadjusted time-effect analysis of full cohort 8

Table S1: List of questions on memory and daily activities and response categories

Questions	Response categories
"Has anyone told you or suggested that you have a poor memory in the past month?"<br><br>"Have you experienced difficulties recalling factual information you previously knew by heart in the past month?"<br><br>"Have you experienced difficulties remembering other people's names in the past month?"	1. No<br>2. Yes, but less than 1 time a week<br>3. Yes, at least 1 time a week<br>4. Yes, at least 3 times a week<br>5. Yes, at least 1 time a day<br>6. Yes, at least 3 times a day<br>7. Yes, more than 3 times a day or all the time
"What proportion of your daily activities have you been able to perform in the past month?"	1. I have not been able to perform any of my daily activities<br>2. I have been able to perform less than half of my daily activities<br>3. I have been able to perform about half of my daily activities<br>4. I have been able to perform more than half of my daily activities<br>5. I have been able to perform all of my daily activities
"How was your ability to perform household tasks, for example cooking, washing up, or cleaning the house, in the past month?"<br><br>"How was your ability to shop for food or other necessities in the past month?"<br><br>"How was your ability to socialise with friends and relatives in the past month?"	1. Not applicable, I usually do not [perform household tasks/ shop/ socialize with friends and relatives]<br>2. No ability to [...]<br>3. Little ability to [...]<br>4. Moderate ability to [...]<br>5. Great ability to [...]

The questions were originally presented in Swedish in the questionnaire.

Table S2a: Frequency of responses to the questions on memory function

		Preoperatively		*4 weeks*		*12 months*	
Question	***Response***	***Control***	***Intervention***	***Control***	***Intervention***	***Control***	***Intervention***
Has anyone told you or suggested that you have a poor memory in the past month?	No	231 (76.7%)	223 (83.8%)	238 (81.5%)	205 (84.4%)	188 (70.9%)	186 (74.7%)
	Yes, <1 time/week	47 (15.6%)	21 (7.9%)	28 (9.6%)	24 (9.9%)	47 (17.7%)	39 (15.7%)
	Yes, 1 time/week	19 (6.3%)	13 (4.9%)	14 (4.8%)	4 (1.6%)	21 (7.9%)	15 (6%)
	Yes, 3 times/week	1 (0.3%)	7 (2.6%)	6 (2.1%)	8 (3.3%)	3 (1.1%)	4 (1.6%)
	Yes, daily[a]	3 (1%)	2 (0.8%)	6 (2.1%)	2 (0.8%)	6 (2.3%)	5 (2%)
	Missing	34	30	43	53	70	47
Have you experienced difficulties recalling factual information you previously knew by heart in the past month?	No	206 (68%)	181 (68.3%)	215 (73.1%)	172 (70.8%)	161 (61.5%)	143 (57.7%)
	Yes, <1 time/week	62 (20.5%)	52 (19.6%)	47 (16%)	41 (16.9%)	54 (20.6%)	74 (29.8%)
	Yes, 1 time/week	27 (8.9%)	23 (8.7%)	18 (6.1%)	21 (8.6%)	29 (11.1%)	22 (8.9%)
	Yes, 3 times/week	3 (1%)	7 (2.6%)	5 (1.7%)	5 (2.1%)	9 (3.4%)	5 (2%)
	Yes, daily[a]	5 (1.7%)	2 (0.8%)	9 (3.1%)	4 (1.6%)	9 (3.4%)	4 (1.6%)
	Missing	32	31	41	53	73	48
Have you experienced difficulties remembering other people's names in the past month?	No	144 (47.2%)	115 (43.1%)	162 (54.7%)	132 (54.3%)	101 (38%)	92 (37.2%)
	Yes, <1 time/week	73 (23.9%)	84 (31.5%)	51 (17.2%)	61 (25.1%)	87 (32.7%)	90 (36.4%)
	Yes, 1 time/week	52 (17%)	37 (13.9%)	44 (14.9%)	28 (11.5%)	42 (15.8%)	41 (16.6%)
	Yes, 3 times/week	21 (6.9%)	17 (6.4%)	18 (6.1%)	13 (5.3%)	16 (6%)	9 (3.6%)
	Yes, daily[a]	15 (4.9%)	14 (5.2%)	21 (7.1%)	9 (3.7%)	20 (7.5%)	15 (6.1%)
	Missing	30	29	39	53	69	49

[a]This response category combines the following responses: Yes, at least 1 time a day; Yes, at least 3 times a day; Yes, more than 3 times a day or all the time

Table S2b: Frequency of responses to the questions on daily activities

		Preoperatively		*4 weeks*		*12 months*	
Question	***Response***	***Control***	***Intervention***	***Control***	***Intervention***	***Control***	***Intervention***
What proportion of your daily activities have you been able to perform in the past month?	None	7 (2.3%)	3 (1.1%)	18 (6.1%)	11 (4.6%)	4 (1.5%)	3 (1.2%)
	Less than half	19 (6.2%)	11 (4.1%)	50 (16.9%)	46 (19.2%)	12 (4.5%)	12 (4.8%)
	About half	22 (7.1%)	20 (7.5%)	54 (18.3%)	37 (15.4%)	14 (5.3%)	14 (5.6%)
	More than half	45 (14.6%)	36 (13.5%)	78 (26.4%)	64 (26.7%)	34 (12.8%)	33 (13.3%)
	All	215 (69.8%)	196 (73.7%)	95 (32.2%)	82 (34.2%)	201 (75.8%)	187 (75.1%)
	Missing	27	30	40	56	70	47
How was your ability to perform household tasks, for example cooking, washing up, or cleaning the house, in the past month?	No ability	1 (0.4%)	2 (0.8%)	17 (6.4%)	5 (2.3%)	1 (0.4%)	1 (0.4%)
	Little ability	17 (6%)	7 (2.9%)	41 (15.4%)	22 (10.1%)	10 (4%)	6 (2.7%)
	Moderate ability	70 (24.6%)	49 (20.4%)	112 (42.1%)	93 (42.7%)	55 (21.8%)	53 (23.7%)
	Great ability	197 (69.1%)	182 (75.8%)	96 (36.1%)	98 (45%)	186 (73.8%)	164 (73.2%)
	Not applicable[a]	22	24	24	22	13	24
	Missing	28	32	45	56	70	48
How was your ability to shop for food or other necessities in the past month?	No ability	4 (1.4%)	4 (1.6%)	30 (11.4%)	13 (6%)	2 (0.8%)	5 (2.2%)
	Little ability	13 (4.5%)	5 (2%)	44 (16.7%)	33 (15.1%)	5 (2.1%)	3 (1.3%)
	Moderate ability	56 (19.4%)	49 (19.7%)	95 (36%)	75 (34.4%)	45 (19%)	45 (20.2%)
	Great ability	215 (74.7%)	191 (76.7%)	95 (36%)	97 (44.5%)	185 (78.1%)	170 (76.2%)
	Not applicable[a]	19	18	27	22	28	24
	Missing	28	29	44	56	70	49
How was your ability to socialise with friends and relatives in the past month?	No ability	2 (0.7%)	4 (1.5%)	12 (4.2%)	16 (6.8%)	6 (2.3%)	3 (1.3%)
	Little ability	24 (7.9%)	24 (9.2%)	57 (20.1%)	41 (17.4%)	20 (7.8%)	16 (6.8%)
	Moderate ability	99 (32.7%)	88 (33.8%)	111 (39.1%)	92 (39.1%)	70 (27.2%)	80 (34%)
	Great ability	178 (58.7%)	144 (55.4%)	104 (36.6%)	86 (36.6%)	161 (62.6%)	136 (57.9%)
	Not applicable[a]	4	5	7	6	6	6
	Missing	28	31	44	55	72	55

[a]Not applicable was removed from analysis

Table S3: Results of the primary intention-to-treat analysis

Odds ratios (OR) and Confidence Intervals (CI) for Intervention vs Control	*Time*	*OR*	*CI Low*	*CI High*	*p value*
Has anyone told you or suggested that you have a poor memory in the past month?[a]	4w	0.91	0.51	1.59	0.74
	12m	0.61	0.38	0.98	0.04
Have you experienced difficulties recalling factual information you previously knew by heart in the past month?[a]	4w	1.16	0.75	1.80	0.51
	12m	0.87	0.59	1.30	0.50
Have you experienced difficulties remembering other people's names in the past month?[a]	4w	0.81	0.55	1.17	0.26
	12m	0.76	0.54	1.09	0.13
What proportion of your daily activities have you been able to perform in the past month?[b]	4w	0.96	0.69	1.33	0.81
	12m	0.80	0.52	1.24	0.33
How was your ability to perform household tasks, for example cooking, washing up, or cleaning the house, in the past month?[c]	4w	1.36	0.94	1.97	0.10
	12m	0.89	0.54	1.44	0.63
How was your ability to shop for food or other necessities in the past month?[c]	4w	1.53	1.07	2.20	0.02
	12m	0.82	0.59	1.30	0.50
How was your ability to socialise with friends and relatives in the past month?[c]	4w	1.00	0.71	1.40	0.98
	12m	0.90	0.60	1.34	0.60

The proportional odds logistic regression model is adjusted for surgical method (laparoscopic or open), tumour location (rectum or colon), and neoadjuvant treatment (no; short-term radiotherapy or long-term chemo- or radiochemotherapy), as well as preoperative values and includes the following response categories:

[a]no; yes, <1 time/week; yes, 1 time/week; yes, 3 times/week; yes, daily
[b]none; less than half; about half; more than half
[c]no ability; little ability; moderate ability; great ability .

Table S4: Results of the per-protocol analysis

Odds ratios (OR) and Confidence Intervals (CI) for Intervention vs Control	*Time*	*OR*	*CI Low*	*CI High*	*p value*
Has anyone told you or suggested that you have a poor memory in the past month?[a]	4w	0.92	0.50	1.66	0.78
	12m	0.61	0.36	1.01	0.06
Have you experienced difficulties recalling factual information you previously knew by heart in the past month?[a]	4w	1.14	0.70	1.82	0.60
	12m	0.84	0.55	1.30	0.45
Have you experienced difficulties remembering other people's names in the past month?[a]	4w	0.90	0.60	1.33	0.59
	12m	0.72	0.48	1.05	0.09
What proportion of your daily activities have you been able to perform in the past month?[b]	4w	1.09	0.77	1.55	0.62
	12m	1.05	0.65	1.73	0.84
How was your ability to perform household tasks, for example cooking, washing up, or cleaning the house, in the past month?[c]	4w	1.49	1.01	2.21	0.05
	12m	1.12	0.65	1.94	0.69
How was your ability to shop for food or other necessities in the past month?[c]	4w	1.59	1.08	2.36	0.02
	12m	1.00	0.55	1.82	1.00
How was your ability to socialise with friends and relatives in the past month?[c]	4w	1.05	0.72	1.51	0.81
	12m	0.98	0.63	1.53	0.93

The intervention group includes individuals reporting aerobic exercise or inspiratory muscle training at least eight days between inclusion and surgery (n = 197).

The proportional odds logistic regression model is adjusted for surgical method (laparoscopic or open), tumour location (rectum or colon), and neoadjuvant treatment (no; short-term radiotherapy or long-term chemo- or radiochemotherapy), as well as preoperative values and includes the following response categories:

[a]no; yes, <1 time/week; yes, 1 time/week; yes, 3 times/week; yes, daily
[b]none; less than half; about half; more than half
[c]no ability; little ability; moderate ability; great ability .

Table S5: Results of the high-risk subgroup per-protocol analysis

Odds ratios (OR) and Confidence Intervals (CI) for Intervention vs Control	*Time*	*OR*	*CI Low*	*CI High*	*p value*
Has anyone told you or suggested that you have a poor memory in the past month?[a]	4w	0.92	0.50	1.66	0.78
	12m	0.63	0.31	1.25	0.19
Have you experienced difficulties recalling factual information you previously knew by heart in the past month?[a]	4w	0.92	0.46	1.80	0.81
	12m	0.62	0.32	1.20	0.15
Have you experienced difficulties remembering other people's names in the past month?[a]	4w	0.70	0.40	1.21	0.20
	12m	0.56	0.31	1.00	0.05
What proportion of your daily activities have you been able to perform in the past month?[b]	4w	0.81	0.49	1.36	0.43
	12m	0.75	0.39	1.43	0.37
How was your ability to perform household tasks, for example cooking, washing up, or cleaning the house, in the past month?[c]	4w	1.52	0.85	2.72	0.16
	12m	0.84	0.41	1.73	0.63
How was your ability to shop for food or other necessities in the past month?[c]	4w	1.53	0.86	2.76	0.15
	12m	0.64	0.29	1.43	0.28
How was your ability to socialise with friends and relatives in the past month?[c]	4w	1.05	0.72	1.51	0.81
	12m	0.67	0.36	1.24	0.20

The high-risk group (n=242) fulfill the two following conditions: (1) age >70 years or ASA classification ≥3 or long neoadjuvant treatment; and (2) low physical activity, ≤2 on the Saltin-Grimby physical activity level scale. Of these the intervention group includes individuals reporting aerobic exercise or inspiratory muscle training at least eight days between inclusion and surgery (n = 93).

The proportional odds logistic regression model is adjusted for surgical method (laparoscopic or open), tumour location (rectum or colon), and neoadjuvant treatment (no; short-term radiotherapy or long-term chemo- or radiochemotherapy), as well as preoperative values and includes the following response categories:

[a]no; yes, <1 time/week; yes, 1 time/week; yes, 3 times/week; yes, daily
[b]none; less than half; about half; more than half
[c]no ability; little ability; moderate ability; great ability .

Table S6: Unadjusted time-effect analysis of full cohort

	Compared times	*OR*	*CI* *Low*	*High*	*p value*
Has anyone told you or suggested that you have a poor memory in the past month?[a]	4w vs preoperatively	0.84	0.57	1.23	0.64
	12m vs preoperatively	2.13	1.47	3.09	<0.001
	12m vs 4w	2.55	1.73	3.76	<0.001
Have you experienced difficulties recalling factual information you previously knew by heart in the past month?[a]	4w vs preoperatively	0.84	0.61	1.15	0.52
	12m vs preoperatively	1.98	1.45	2.70	<0.001
	12m vs 4w	2.37	1.71	3.28	<0.001
Have you experienced difficulties remembering other people's names in the past month?[a]	4w vs preoperatively	0.67	0.51	0.87	0.01
	12m vs preoperatively	1.44	1.10	1.87	0.02
	12m vs 4w	2.16	1.64	2.85	<0.001
What proportion of your daily activities have you been able to perform in the past month?[b]	4w vs preoperatively	0.13	0.10	0.17	<0.001
	12m vs preoperatively e	1.27	0.94	1.73	0.26
	12m vs 4w	9.79	7.18	13.34	<0.001
How was your ability to perform household tasks, for example cooking, washing up, or cleaning the house, in the past month?[c]	4w vs preoperatively	0.13	0.09	0.18	<0.001
	12m vs preoperatively	1.12	0.81	1.57	0.77
	12m vs 4w	8.91	6.25	12.69	<0.001
How was your ability to shop for food or other necessities in the past month?[c]	4w vs preoperatively	0.10	0.07	0.14	<0.001
	12m vs preoperatively	1.12	0.79	1.57	0.81
	12m vs 4w	11.74	8.10	17.03	<0.001
How was your ability to socialise with friends and relatives in the past month?[c]	4w vs preoperatively	0.27	0.20	0.35	<0.001
	12m vs preoperatively	1.15	0.87	1.52	0.59
	12m vs 4w	4.29	3.22	5.73	<0.001

The proportional odds logistic regression model is unadjusted and includes the following response categories:

[a]no; yes, <1 time/week; yes, 1 time/week; yes, 3 times/week; yes, daily

[b]none; less than half; about half; more than half

[c]no ability; little ability; moderate ability; great ability
