# Supplementary material for: Effect of exercise on memory function and daily activities after colorectal cancer surgery: an exploratory secondary analysis from the randomised trial PHYSSURG-C
Source: BMJ Open. 2026 Sep 23;16(9):e120096. doi: 10.1136/bmjopen-2026-120096 (PMC13630044; doi:10.1136/bmjopen-2026-120096)
Supplement: Supplementary file 2 [file bmjopen-16-9-s002.pdf]

Statistical Analysis Plan

PHYSSURG-C 12 months
Paper III

Memory function and daily activities

VERSION 3.1

2024-11-13

Carolina Ehrencrona, Eva Angenete, Kevin Afshari, Sandra Buratti, Charlotta Larsson, Ying Li, Aron Onerup, Anette Wedin, Eva Haglind

Amendment 2025-02-07
Adjustment variables for cancer site is changed from the registry variable A2_tumlok to being based on the variable Type from the inclusion CRF due to missing in A2_tumlok.

INTRODUCTION

This Statistical Analysis Plan (SAP) details the statistical and data-related aspects of a planned analysis on the effects on cognitive function as well as some aspects of functions of activities of daily life. The setting is a randomized trial of an intervention consisting of added aerobic physical activity in connection with surgery for colorectal cancer (PHYSSURG-C). The time is up to 1-year follow-up after surgery. The primary analyses will be between the two randomized groups, that is by intention to treat, and we will consider a secondary set of analyses where groups should be divided by exposure (level of physical activity) prior to randomization in an observational study.

Level of physical activity has been reported by WHO to influence risk of developing certain types of cancer, one being colorectal cancer. For patients diagnosed with colorectal cancer prehabilitation before treatment has been hypothesized to improve outcome, but studies have reached different results[1,2,3], also regarding frail patients[4,5]. In two of our previous studies and in other studies with different designs, it has been suggested that individuals with high/very high levels of habitual physical activity before diagnosis, had a faster recovery and/or decreased risk of complications after treatment, mainly surgery[6,7,8]. In PHYSSURG-C were increased aerobic physical activity to the level recommended by WHO before surgery and after discharge was compared with routine care, we did not find any difference in primary outcome: physical recovery at 4 weeks[3]. When regarding the trial as an observational cohort, the level of habitual physical activity before diagnosis was associated with risk of postoperative complications[8].

It has been reported that anesthesia during surgery can affect cognitive function[9]. This could in part be due to effects of pharmacological agents used in anesthesia, but the surgical trauma as such could contribute. It has been clinically recognized for a long time that persons with slight, early signs of cognitive dysfunction can become severely affected after an operation. The condition is however often short in duration and reversible[9]. Meta-analyses aiming to differentiate the effect from different anesthesiologic agents were inconclusive due to lack of high quality trials[12,13] as was a recent randomized trial comparing two anesthesiologic agents[14]. Risk factors for post-chemotherapy cognitive dysfunction in patients with colorectal cancer include low level of exercise, high BMI and old age, among others[10]. A recent review summarized that cognitive impairment could occur after several types of treatment and that risk factors also include genetic factors as well as early signs of neurological changes[11]. The review also highlights the possibility of physical exercise as an intervention. In a review, McDonald found that structural changes in white and grey matter could be associated with cancer as well as therapies against cancer[15]. In a study of cognitive dysfunction in

Postadress
Sahlgrenska universitetssjukhuset
SSORG
416 85 Göteborg

connection with treatment of prostate cancer, the strongest association was reported with hormonal therapy[16]. Impaired function in activities of daily living is a diagnostic criterium for neurocognitive disorders[17] and an indication of subtle changes in cognitive function[18].

The primary aim of this study is to analyze effects on subjective memory function at 4 weeks and 12 months following surgery for colon or rectal cancer and self-reported function of daily life as secondary aim.

ANALYSIS OBJECTIVES

Objectives are to evaluate the effect of the intervention mentioned above on the following secondary outcome measures by comparing outcomes between intervention and control groups:

- Memory difficulties as reported by the participants
- Functions of daily life reported by the participants

The difference between intervention and control group will be reported as at 4 week, 12 months and as combined follow-up.

METHODS

The PHYSSURG-C trial was designed as a randomized, controlled, multi-center open-label trial comparing an intervention of pre- and postoperative physical activity with standard care regarding postoperative recovery after colorectal cancer surgery. Since the methods have been described previously[3], a brief summary of design is given here.

The design was prospective, randomized, open-label, multicenter in Sweden. Sample size was calculated based on a superiority hypothesis regarding the effect of the intervention studied on the primary outcome, physical recovery at 4 weeks after surgery. A total of six hospitals included patients. Randomization was stratified by tumor location, neoadjuvant treatment and surgical method. The Regional Ethics Board in Gothenburg (2014-10-30, DNR:597-14) gave permission and registration at ClinicalTrials.gov (NCT02299596) was made before inclusion of the first patient.

Data retrieval through CRF was made by study nurses at inclusion and from patients through questionnaires at baseline as well as at 4 weeks and 12 months. From the Swedish ColoRectal Cancer Register (SCRCR), pTNM, ASA, radical/not surgery, open/laparoscopic technique were retrieved. pTNM was used to calculate tumor stage. Clinical outcomes within 12 months were retrieved from hospital records by two of the authors using prespecified CRFs.

Postadress
Sahlgrenska universitetssjukhuset
SSORG
416 85 Göteborg

Questionnaires were in the analogue form, and given to each included patient at baseline, to be filled out at home and returned by pre-paid envelope. At follow-up, a study nurse contacted each patient by phone before sending out 4 weeks or 12 months questionnaires respectively. One remainder letter was sent 2 weeks after sending the questionnaire. Questionnaires were returned to the secretariate using a pre-paid envelope.

Inclusion criteria Age 20 years or more; elective surgery with curative intent for colon or rectal cancer; understanding Swedish. Patients were informed and asked for consent at diagnosis or during the same week as diagnosis.

Exclusion criteria Emergency surgery, local excision, cytoreductive surgery with HIPEC, inability to perform the intervention (physical inability or short time until planned surgery).

Sample size was calculated before start of the inclusion, but due to the restricted knowledge of what to expect regarding effect size, a planned interim analysis by an external group was made after the first 100 randomizations, to recalculate the sample size. The resulting sample size was submitted to the Ethical board and accepted. One further increase of the sample size was made at a later stage when it was obvious that drop out was larger than expected.

ANALYSIS POPULATION, TYPE OF ANALYSIS

Analysis population

The analysis population consists of all randomized participants who remained through the 12 month follow up.

Type of analysis

The primary analyses will be as randomized, that is intention-to-treat analysis.

For exploratory reasons we will also perform per-protocol analyses. In these analyses, participants randomized to the intervention group who have returned exercise diaries and reported preoperative exercise (inspiratory muscle training and/or aerobic exercise) during at least eight days, and at least 80% of possible days between inclusion and surgery, will be analyzed as having performed the intervention. All participants randomized to control will be analyzed as control. Participants randomized to intervention but who have not returned exercise diaries, or who have returned diaries not meeting the criteria for compliance described above will be excluded from the per protocol analyses.

A third set of analyses will be considered, namely by exposure to physical activity before randomization, that is mirroring the physical activity habits of the individual in an observational study and not assessing the effect of the intervention.

Postadress
Sahlgrenska universitetssjukhuset
SSORG
416 85 Göteborg

VARIABLES AND ENDPOINTS

- **Memory difficulties**
  - “Has anyone pointed out or suggested that you have a poor memory during the last month?”
  - “Have you experienced difficulties remembering other people’s names during the last month?”
  - ”Have you experienced difficulties remembering factual knowledge you have previously known by heart during the last month?”

  At 4 weeks (Q1Q68-70) and 12 months (Q2Q75-Q77). Baseline values will also be presented and adjusted for (Q0A113-A115).

- **Functions of daily life**
  - “What proportion of your daily activities have you managed to perform during the last month?”
  - “How was your ability to perform household tasks, for example to cook, to wash up, or to house clean during the last month?”
  - “How was your ability to shop for example food or other necessities during the last month?”
  - “How was your ability to socialize with friends and relatives during the last month?”

  At 4 weeks (Q1Q76, Q78-80) and 12 months (Q2Q82, Q84-86). Baseline values will also be presented and adjusted for (Q0A123, Q0A125-A127).

Covariates, stratum, and subgroups

Since PHYSSURG-C is a randomized, controlled trial adjustment for covariables in the primary analysis is restricted since any variation in confounders is assumed to occur at random.

We will adjust for stratification variables:

- Colon cancer or rectal cancer from (Type), with neoadjuvant (chemo)radiotherapy or without neoadjuvant (chemo)radiotherapy from the hospital records (Neoadjuvant_therapy).
  Open or laparoscopic surgery, retrieved from the registry (opmod).

Adjustment for baseline value will be made for the outcome variables: memory difficulties (Q0A113-A115), activities of daily life (Q0A123, Q0A125-A127).

Postadress
Sahlgrenska universitetssjukhuset
SSORG
416 85 Göteborg

Subgroups
Intervention with pre- and postoperative physical activity is likely to have its largest effect in patients who have a high risk for prolonged recovery in impairment of memory or functions of daily life and who are habitually relatively physically inactive[4-6]. A supporting subgroup analyses will therefore be performed for old, inactive, or frail patients defined by fulfilling the two following conditions:

1. Age >70 years old or ASA classification ≥3(A2_asaklass) or long preoperative (chemo)radiotherapy(neoadjuvant_therapy)
2. Saltin-Grimby (Q0A97) ≤2

Sensitivity
Sensitivity analysis to explore if outcome is mediated by adjusted factors will be made for:

Primary outcome, memory difficulties, with adjustment for depressed mood (Q1Q62, Q2Q69) and worry/anxiety (Q1Q63, Q2Q70).

Secondary outcome, function of daily activities, with adjustment for physical stamina (Q1Q61, Q2Q68).

HANDLING OF MISSING VALUES AND OTHER DATA CONVENTIONS

A dropout analysis was performed in the primary analyses (4 weeks physical recovery) of this trial[8].

When there is at least one missing value for the factors in a multiple regression model, the default approach is listwise deletion where the entire record is excluded from analysis if any single value is missing. No imputation of missing values will be made.

For missing values of outcome variables sensitivity analyses will be considered.

STATISTICAL METHODOLOGY

General principles

The analyses of memory difficulties and function of daily activities should be regarded as exploratory and no corrections for multiplicity will be made.

Type of analyses

The primary analysis will be an analysis adjusted for the stratification factors listed above. We will also report unadjusted analyses as supporting analysis.

Postadress
Sahlgrenska universitetssjukhuset
SSORG
416 85 Göteborg

Sample size

Sample size was calculated in relation to the primary endpoint for the trial, physical recovery at 4 weeks postoperatively. In order to achieve 80% power to detect difference using a two-sided test with 5% significance level, a total of 538 evaluable patients were required. We planned to include 640 participants to compensate for loss to follow-up. However, when around 600 patients had been included, it was obvious that loss to follow-up was larger than anticipated. Due to this, inclusion continued to 780 randomized participants[8].

Of the 668 randomized and eligible participants 37 withdrew consent before the 12-month follow-up. The population for this analysis is 631 (see flowchart p. 9).

Demography and patient characteristics

For Table 1 the following information is intended to be presented.

- **Age** (age). Mean, SD.
- **BMI** (bmi). Mean, SD.
- **Sex** (gender). Male/female. Frequency female (%).
- **Education** (utbildning). Frequency (%) Compulsary, Upper secondary, University.
- **Ethnicity** (Etnicitet). Frequency born in Sweden (%).
- **Occupation** (Occupation). Frequency (%).
- **Marital status** (Marital). Dichotomized as Married or cohabiting (1, 3-5, 7), single household (2-6). Frequency (%) Baseline.
- **Smoking** (smoking). Dichotomized into current regular smoker (4 & 5) or not (1, 2 & 3). Presented as frequency current regular smoker (%). (Baseline)
- **Alcohol** (AuditC_score). Presented as Mean, SD. (Baseline)
- **Level of physical activity** (Q0A97). Frequency for each category (%). (Baseline)
- **ASA** classification. Retrieved from The Swedish Colorectal Cancer Registry (A2_asaklass). Frequency for each category (%).
- **Comorbidity** (Q0A19X, Q0A20). See addendum (Baseline)
- **Tumor stage** calculated from pTNM classification, collected from The Swedish Colorectal Cancer Registry (stage). Frequency stage for each stage (1, 2, 3 & 4) (%).
- **Type of performed surgery**. Retrieved from The Swedish Colorectal Cancer Registry (optyp). Categorized as any form of colectomy (=02-06), rectum resection (=07), or abdominoperineal excision of rectum (=08). Frequency (%).
- **Open or laparoscopic surgery**, retrieved from The Swedish Colorectal Cancer Registry (opmod). Frequency (%).
- **Postoperative delirum at index stay**(confusion) Frequency (%).
- **Adjuvant therapy** (Neoadjuvant, Adjuvant) Frequency (%)

Postadress
Sahlgrenska universitetssjukhuset
SSORG
416 85 Göteborg

Primary and secondary endpoints

All primary outcomes variables have the same alternatives. The three highest levels will be used as one category (i.e. daily).

1. No
2. Yes, but less often than 1 time a week
3. Yes, at least 1 time a week
4. Yes, at least 3 times a week
5. Yes, at least 1 time a day
6. Yes, at least 3 times a day
7. Yes, more than 3 times a day or all the time

The secondary outcome "What proportion of your daily activities have you managed to perform during the last month?" has the following alternatives which will all be used in the model:

1. I have not managed to perform any of my daily activities
2. I have managed to perform less than half of my daily activities
3. I have managed to perform about half of my daily activities
4. I have managed to perform more than half of my daily activities
5. I have managed to perform all my daily activities

The remaining secondary outcomes all have the same alternatives. Not applicable will be removed from analysis. The remaining levels will be preserved in the model.

1. Not applicable
2. No ability
3. Little ability
4. Moderate ability
5. Great ability

The proportional odds assumption for the categories in each outcome will be statistically tested at 5% level.

If the null hypothesis of the assumption is not rejected, a proportional odds model will be used. Results will be presented as odds ratio (intervention vs control), 95% confidence intervals (95% CI) and a p-value. Group (as randomized) and adjustment factors will be included as fixed effects.

If the proportional odds assumption is not fulfilled, a multinomial logistic or quantile regression will be used. If the assumptions behind the parametric analyses are seriously violated non-parametric methods will be used.

Postadress
Sahlgrenska universitetssjukhuset
SSORG
416 85 Göteborg

TECHNICAL DETAILS

Software used are:

- SAS®,
- R®.

LIST OF TABLES AND FIGURES PLANNED FOR THE MANUSCRIPT

Figure 1: Flow chart.

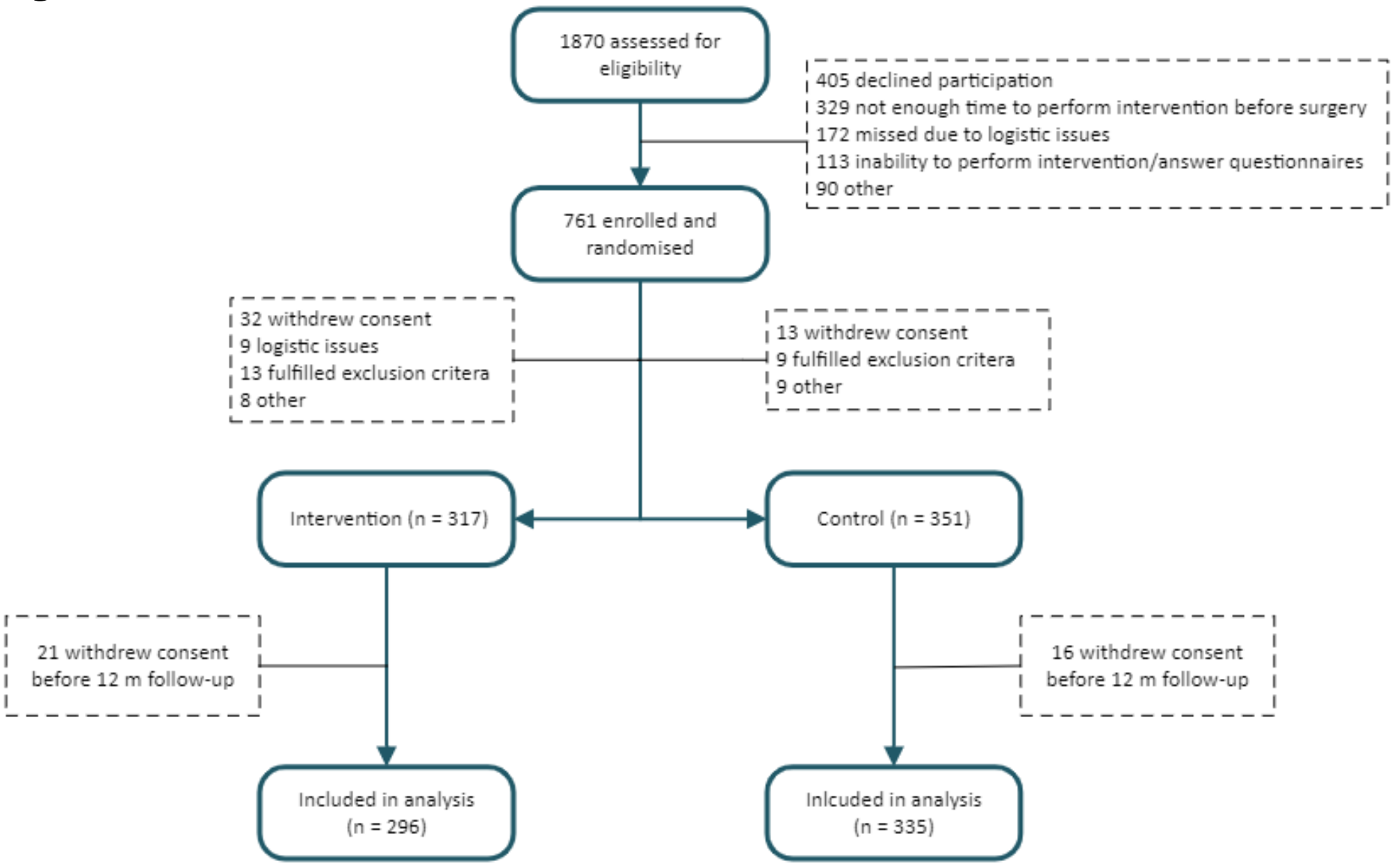

Figure 2: Results (If applicable).

Table 1: Demography, including tumor and surgical details (see above) and characteristics at baseline for 12 months cohort.

Table 2: Adjusted analyses memory difficulties (a) and function of daily life (b).

Values for all endpoints will also be presented either as graphs or tables within manuscript or in supplement.

Postadress
Sahlgrenska universitetssjukhuset
SSORG
416 85 Göteborg

REFERENCES

1. Kennedy RH, Francis EA, Wharton R, et al. Multicenter randomized controlled trial of conventional versus laparoscopic surgery for colorectal cancer within an enhanced recovery programme: EnROL. J Clin Oncol. 2014;32:1804– 1811

2. Gustafsson UO, Scott MJ, Hubner M, et al. Guidelines for perioperative care in elective colorectal surgery: enhanced recovery after surgery (ERAS((R))) society recommendations: 2018. World J Surg. 2019;43:659–695

3. Onerup A, Andersson J, Angenete E, Bock D, Börjesson M, Ehrencrona C, Fagevik Olsén M, Larsson PA, de la Croix H, Wedin A, Haglind E. Effect of short-term homebased pre- and postoperative exercise on recovery after colorectal cancer surgery (PHYSSURG-C): A randomized clinical trial. Ann Surg. 2022 Mar 1;275(3):448-455.

4. Barberan-Garcia A, Ubre M, Roca J, et al. Personalised prehabilitation in high-risk patients undergoing elective major abdominal surgery: a randomized blinded controlled trial. Ann Surg. 2018;267:50–56

5. Carli F, Bousquet-Dion G, Awasthi R, et al. Effect of multimodal prehabilitation vs postoperative rehabilitation on 30-day postoperative complications for frail patients undergoing resection of colorectal cancer: a randomized clinical trial. JAMA Surg. 2020;155:233–242

6. Onerup A, Bock D, Borjesson M, et al. Is preoperative physical activity related to post-surgery recovery?-a cohort study of colorectal cancer patients. *Int J Colorectal Dis* 2016;31(6):1131-40. doi: 10.1007/s00384-016-2551-4

7. Onerup A, Angenete E, Bonfre P, et al. Self-assessed preoperative level of habitual physical activity predicted postoperative complications after colorectal cancer surgery: A prospective observational cohort study. *Eur J Surg Oncol* 2019;45(11):2045-51. doi: 10.1016/j.ejso.2019.06.019 [published Online First: 2019/06/21]

8. Onerup A, Angenete E, Bock D, et al. Association between self-assessed preoperative level of physical activity and postoperative complications - An observational cohort analysis within a randomized controlled trial (PHYSSURG-C). *Eur J Surg Oncol* 2022;48(4):883-89. doi: 10.1016/j.ejso.2021.10.033

9. Bedford PD. Adverse cerebral effects of anaesthesia on old people. Lancet. 1955 Aug 6;269(6884):259-63.

Postadress
Sahlgrenska universitetssjukhuset
SSORG
416 85 Göteborg

10. Zhou SP, Fei SD, Han HH, Li JJ, Yang S, Zhao CY. A Prediction Model for Cognitive Impairment Risk in Colorectal Canc er after Chemotherapy Treatment..Biomed Res Int. 2021 Feb 20;2021:6666453.

11. Lange M , Joly , Vardy J , Ahles T , Dubois M , Tron L, Winocur G , De Ruiter MB , Castel H. Cancer-related cognitive impairment: an update on state of the art, detection, and management strategies in cancer survivors. Ann Oncol; 2019 Dec 1;30(12):1925-1940.

12. Miller D, Lewis SR, Pritchard MW, Schofield-Robinson OJ, Shelton CL, Alderson P, Smith AF. Intravenous versus inhalational maintenance of anaesthesia for postoperative cognitive outcomes in elderly people undergoing non-cardiac surgery. Cochrane Database of Systematic Reviews 2018, Issue 8. Art. No.: CD012317. DOI: 10.1002/14651858.CD012317.pub2.

13. Herling SF, Dreijer B, Wrist Lam G, Thomsen T, Møller AM. Total intravenous anaesthesia versus inhalational anaesthesia for adults undergoing transabdominal robotic assisted laparoscopic surgery. Cochrane Database of Systematic Reviews 2

14. Yujuan Li, Dongtai Chen, Hanbing Wang, Zhi Wang, Furong Song, Hui Li, Li Ling, Zhiwen Shen, Chuwen Hu, Jun Peng, Weixing Li, Wei Xing, Jiahao Pan, Hua Liang, Qiaoling Zhou, Jun Cai, Ziqing He, Shuling Peng, Weian Zeng, Zhiyi Zuo. Intravenous versus Volatile Anesthetic Effects on Postoperative Cognition in Elderly Patients Undergoing Laparoscopic Abdominal Surgery. Anesthesiology. 2021 Mar 1;134(3):381-394.

15. McDonald BC. Structural Neuroimaging Findings Related to Adult Non-CNS Cancer and Treatment: Review, Integration, and Implications for Treatment of Cognitive Dysfunction. Neurotherapeutics (2021) 18:792–810.

16. Reiss AB , Saeedullah U, Grossfeld DJ , Glass AD , Pinkhasov A , Katz AE. Prostate cancer treatment and the relationship of androgen deprivation therapy to cognitive function. Clin Transl Oncol. 2022; May;24(5):733-741.

17. American Psychiatric Association D. S. M. Task Force. Diagnostic and statistical manual of mental disorders : DSM-5. Fifth edition ed. Washington, DC: Washington, DC: American Psychiatric Publishing; 2013.

Postadress
Sahlgrenska universitetssjukhuset
SSORG
416 85 Göteborg

18. Evered SL, Silbert AB, Knopman TD, Scott SD, Dekosky SS, Rasmussen GL, et al. Recommendations for the Nomenclature of Cognitive Change Associated with Anaesthesia and Surgery—2018. Anesthesiology. 2018;129(5):872-9.

Addendum

Q0A19X comorbidity should be classified as
Cardiovascular=2+4+5+6+7
Neurologic = 1+3
Pulmonary (COPD)= 8
Psychiatric(mental) =11+12+13
Chronic pain=14
Others=9+10+15+16

Q0A20
Diabetes – yes/no

Postadress
Sahlgrenska universitetssjukhuset
SSORG
416 85 Göteborg
