# Supplementary material for: Effect of exercise on memory function and daily activities after colorectal cancer surgery: an exploratory secondary analysis from the randomised trial PHYSSURG-C
Source: BMJ Open. 2026 Sep 23;16(9):e120096. doi: 10.1136/bmjopen-2026-120096 (PMC13630044; doi:10.1136/bmjopen-2026-120096)
Supplement: Supplementary file 3 [file bmjopen-2026-120096.reviewer_comments.pdf]

PEER REVIEW HISTORY

BMJ Open publishes all reviews undertaken for accepted manuscripts. Reviewers are asked to complete a checklist review form and are provided with free text boxes to elaborate on their assessment. These free text comments are reproduced below.

ARTICLE DETAILS

Title (Provisional)

Effect of exercise on memory function and daily activities after colorectal cancer surgery - An exploratory secondary analysis from the randomised trial PHYSSURG-C

Authors

Ehrencrona, Carolina; Li, Ying; Afshari, Kevin; Angenete, Eva; Buratti, Sandra; Larsson, Charlotta; Onerup, Aron; Wedin, Anette; Haglind, Eva

---

VERSION 1 - REVIEW

**Reviewer**	**1**
**Name**	**Lau, Jerrald**
**Affiliation**	**National University of Singapore, Saw Swee Hock School of Public Health**
**Date**	**16-Jun-2026**
**COI**	**None**

This was a succinctly written manuscript describing exploratory analyses resulting from the PHYSSURG-C trial, pertaining to differences between the intervention and control groups on self-reported memory function as well as ADLs. I do have some minor comments and suggestions for the authors' consideration:

Introduction

- (Page 4, lines 69-75) Is there any existing evidence of long term effects on cancer-related cognitive impairment from behavioural interventions? A short summary of these prior findings (if any) would help to justify more strongly the rationale for this study.

- (Page 4, line 80) Why was 12 months post-surgery chosen as the endpoint? I assume this is linked to the original PHYSSURG-C's design, but I think a brief explanation here or in the Methods would be helpful.

Results

- (Page 7, line 167; Figure 1) Mentioning the number of participants who were per-protocol is helpful, but this can be even clearer if this breakdown was included somewhere in the study flowchart.

Discussion

- (Page 10, lines 238-239) I agree with the comment on multiple testing. My question then, is why not present the findings corrected for multiple testing? That would allow you to comment further on the adjusted versus original analyses.

Other comments

- (Overall, throughout manuscript) Some additional proofreading is needed to address sentence structure as well as use of informal language, e.g., "it's"

**Reviewer**	**2**
**Name**	**Rydwik, Elisabeth**
**Affiliation**	**Karolinska Institute, Neurobiology, Care Sciences and Society**
**Date**	**29-Jun-2026**
**COI**	**None**

General comments

The manuscript is well written, and the outcomes cognition and ADL are of importance even though this study was not preplanned as far as I can understand and the data are secondary and exploratory. My main comments relate to the methods, questionnaires used and statistical analysis.

Specific comments

Abstract

Please add that the manuscript reports secondary data, not only that they are exploratory.

Introduction

Please, provide a rational for evaluating ADL, this is lacking in the introduction. In the discussion you state the following "…and the ability to perform daily activities and specific activities provides only an indirect indication of cognitive functioning abilities.". This should be elaborated on in the introduction.

I also lack a rational for why you used the whole sample for this type of analyses when the inclusion criteria was 20 and above, even though you conducted a sensitivity analysis on an older age group. Cognitive problems occur mainly in older age groups, please explain why you chose to include the whole sample.

Methods

The questionnaires in this study were not listed in the study protocol, please, explain why. When you explain how the questionnaires were created you refer to a paper that was published in 2024, but the questionnaire must have been created before the study started, please clarify.

According to my understanding the intervention/control group (binary variable) was the dependent variable, please explain why you used a proportional logistic regression model.

You might consider rewriting this sentence, "..including the per-protocol population of all participants randomised to the control group…" since it might be interpreted as that only a proportion of the control group were used in the per-protocol analysis. I understand that that is not the case but the sentence can be improved for clarity.

Please, state a rational for why you did time-effect-analysis on the whole sample. This must be explained.

Results

Table S2a: It is somewhat surprising that the variable poor memory is significantly different between groups but not difficulty recalling information as the differences between groups are larger for that variable at 12 months. Please elaborate on this in the discussion.

You state in the statistical plan, that you planned to control for depression and mood in the sensitivity analyses, but this is not stated in supplementary Table S4, please explain why.

Discussion

Your conclusion is valid based on design and methodology and the secondary analysis and you discuss most of the relevant limitations such as not using valid instruments for the outcomes.

VERSION 1 - AUTHOR RESPONSE

Reviewer: 1
Dr. Jerrald Lau, National University of Singapore

Comments to the Author:
This was a succinctly written manuscript describing exploratory analyses resulting from the PHYSSURG-C trial, pertaining to differences between the intervention and control groups on self-reported memory function as well as ADLs. I do have some minor comments and suggestions for the authors' consideration:

Introduction

- **(Page 4, lines 6G-75) Is there any existing evidence of long term effects on cancer-related cognitive impairment from behavioural interventions? A short summary of these prior findings (if any) would help to justify more strongly the rationale for this study.**

Studies with long-term follow-up of physical activity interventions for cancer-related cognitive impairment are underrepresented and the evidence is unclear. We have added information on this in the introduction (lines 72-75) :

However, recent reviews have found that evidence regarding the effects of physical activity on cancer-related cognitive difficulties is inconclusive for both short and long-term effects[12, 13]. *Studies of long-term effects were underrepresented, and most assessments of cognitive function occurred at the end of intervention*.

- (Page 4, line 80) Why was 12 months post-surgery chosen as the endpoint? I assume this is linked to the original PHYSSURG-C's design, but I think a brief explanation here or in the Methods would be helpful.

Yes, the 12-month endpoint was part of the original study design, and this has been clarified under Outcomes in the Method section (lines 120-121).

Outcome data were obtained from questionnaires administered at the 4-week and 12-month follow-ups in the original trial.

The reason for 12 months was that at the time of finalising the protocol for PHYSSURG-C, studies and trials on physical prehabilitation measured effects at the time of operation or at most within 1-2 months after cancer surgery. As all patients included in the trial had surgery with curative intentions, and a longer time perspective was considered meaningful. One year is a common time for postoperative measurement in clinical studies and also follow-up in routine care.

Results

- (Page 7, line 167; Figure 1) Mentioning the number of participants who were per-protocol is helpful, but this can be even clearer if this breakdown was included somewhere in the study flowchart.

Thank you for the suggestion. The flowchart has been edited to accommodate the per-protocol and subgroup populations. See figure 1.

Discussion

- (Page 10, lines 238-23G) I agree with the comment on multiple testing. My question then, is why not present the findings corrected for multiple testing? That would allow you to comment further on the adjusted versus original analyses.

This analysis was secondary and exploratory in nature, and the aim was not to make conclusive claims. As prespecified in the statistical analysis plan prior to the analyses, no adjustment for multiple comparisons was planned.

Because the analyses were exploratory, applying a multiple-comparison correction would not alter the exploratory nature of the findings or increase the certainty of the conclusions. In this context, we considered it more appropriate to present the prespecified unadjusted results and interpret them cautiously.

We have revised the manuscript to clarify that no correction for multiple testing was applied because of the exploratory nature of the analyses and to further emphasize that the findings should be interpreted with appropriate caution.

For example, the in the statistics section (lines 143-144) and discussion (lines 248-249) respectively:

…these analyses were regarded as exploratory without the intention of making confirmatory claims. Therefore, no correction for multiple testing was applied.

However, the exploratory nature of the analyses and multiple testing without correction, as well as the distribution of odds ratios, warrants caution regarding the interpretation of the isolated significant p-values observed.

Other comments

- **(Overall, throughout manuscript) Some additional proofreading is needed to address sentence structure as well as use of informal language, e.g., "it's"**

Reviewer: 2
Dr. Elisabeth Rydwik, Karolinska Institute, Stockholm County Council

Comments to the Author:
General comments
The manuscript is well written, and the outcomes cognition and ADL are of importance even though this study was not preplanned as far as I can understand and the data are secondary and exploratory. My main comments relate to the methods, questionnaires used and statistical analysis.

Specific comments Abstract
Please add that the manuscript reports secondary data, not only that they are exploratory.

This has been added to the Conclusion part of the abstract:

This exploratory study with secondary data, nested in a multicentre randomised trial, …

Introduction
Please, provide a rational for evaluating ADL, this is lacking in the introduction. In the discussion you state the following "…and the ability to perform daily activities and specific activities provides only an indirect indication of cognitive functioning abilities.". This should be elaborated on in the introduction.

The second paragraph in the introduction was intended to explain the relationship between cognitive functions and daily activities. This has now been revised, and we think it provides a rationale for evaluation of daily activities.

Subjective complaints from cancer patients on decline in cognitive function and the impact on daily life usually concern memory and executive function [5]. A decline in executive function can require increased efforts to resume activities after interruption, plan ahead, make decisions, and follow conversations [c]. Both memory and executive function are linked to the ability to perform daily activities, especially instrumental activities of daily living, such as household chores and shopping [7]. A reduced ability to perform daily activities can be a sign of cognitive decline [8].

I also lack a rational for why you used the whole sample for this type of analyses when the inclusion criteria was 20 and above, even though you conducted a sensitivity analysis on an older age group. Cognitive problems occur mainly in older age groups, please explain why you chose to include the whole sample.

While it is true that postoperative and cancer-related cognitive dysfunction is more common in the elderly, it has been reported to be present at all ages. As this was a pragmatic trial with the intention of investigating an intervention for *all* patients with colorectal cancer undergoing curative intended surgery, we wanted to stay true to that and not primarily analyse a subsample.

As for the sample, the median age is 70 years, and the lower quartile is 62, so it is already mostly an older age group. As it is a randomized trial, the younger patients are also distributed evenly between the groups. However, as we assumed that the intervention could be most effective in elderly or frail and inactive we did a subgroup analysis with the same criteria as the trial's

primary endpoint, as also there it was hypnotized that the intervention would be less effective in younger and fitter patients.

Methods

The questionnaires in this study were not listed in the study protocol, please, explain why. When you explain how the questionnaires were created you refer to a paper that was published in 2024, but the questionnaire must have been created before the study started, please clarify.

All outcome measures for PHYSSURG-C are listed on the trial registration on clinicaltrials.gov. Several established instruments were used in the questionnaires which are the ones listed in the published protocol article. The outcomes presented in this report are however not from an instrument but are within the set described on clinicaltrials.gov as "Health related QoL" that are developed within the study group.

The basis for these questions is interviews with patients and qualitative content analysis, followed by expert and patient validation. Once a set a question has been formulated for a theme, we reuse these items if the same theme is identified in another patient group. The reference to the 2024 paper (Reinwalds et al.) was chosen because of the detailed description of the procedure of developing questionnaires within our group.

In addition to the 2024 paper, we now also reference two protocol papers of cohort studies within our research group that included these outcomes, used the same methodology for making questionnaires and had inclusion prior to PHYSSURG-C. However, neither of these studies have reported on these specific outcomes yet. The questionnaires are extensive covering a multitude of aspects within quality of life, and analyses are ongoing.

To make the origin of the outcome measures clearer we have revised the description of outcomes in the Methods section:

These questions were originally developed by the research group to be used in cohort studies on quality of life in cancer patients[18-20]. The questions were constructed after content analysis of semi-structured patient interviews, and then underwent expert and patient face validation, (see Reinwalds et al., 2024[15] for more detailed description of the procedure).

And rephrased a sentence in the limitations section of the discussion:

Also, the outcomes are based on questions developed by our group that have not been validated against other instruments or measurements.

According to my understanding the intervention/control group (binary variable) was the dependent variable, please explain why you used a proportional logistic regression model.

Proportional odds logistic regression was used as the outcome variables were ordinal scales. While ordinal scales in some contexts are used as continues variables, we did not find it appropriate here as the data was skewed to one end of the scale. To retain a high level of information, we chose to keep as many steps on the scale as possible. We only combined answer alternatives with very few responses to avoid converging problems with the model. We refrained from using a binary cut off as this becomes a rough measurement, and also arbitrary as there is no clear line of what would define important memory problems or interference of daily activities.

You might consider rewriting this sentence, "..including the per-protocol population of all participants randomised to the control group…" since it might be interpreted as that only a proportion of the control group were used in the per-protocol analysis. I understand that that is not the case but the sentence can be improved for clarity.

Thank you for highlighting this. We have rephrased the section:

A per-protocol analysis was performed on the participants in the intervention group who reported aerobic exercise or inspiratory muscle training at least 8 days, between inclusion and surgery, i.e., no less than 80% of the minimum (i.e. 14 - 4) available days. The remaining participants in the intervention arm were excluded while all participants in the control arm were included in the analysis.

Please, state a rational for why you did time-effect-analysis on the whole sample. This must be explained.

The time-effect analysis was performed to explore whether the outcome measures could detect changes over time. If the measurements had been too crude and thus insensitive to detect change, it would be difficult to interpret the absence of an intervention effect. As this was a post hoc analysis, and it had already been established that there was no substantial difference between the groups, we considered this as evaluating the measurements rather than exploring the research question on the effect of the intervention.

The paragraph describing the post hoc analysis has been revised to clarify the rationale:

Further, a post hoc time-effect analysis with an unadjusted proportional odds logistic regression model was carried out for descriptive purposes. This was done to examine if there were any detectable changes in the outcomes over time as we reasoned that if there were no changes it would be unlikely that the intervention could have shown any effect. As this was a post hoc analysis intended to evaluate the measurement, and not compare groups, the whole cohort was pooled in the analysis.

Results

Table S2a: It is somewhat surprising that the variable poor memory is significantly different between groups but not difficulty recalling information as the differences between groups are larger for that variable at 12 months. Please elaborate on this in the discussion.

The statistical model we used compares differences across the entire scale and not within individual levels. Regarding poor memory, all the proportions for all Yes-levels are lower, or comparable, in the intervention group versus the control group making a consistency in the difference between the groups. In contrast, for the recalling information variable, one of the Yes-levels has a considerable lager proportion in the interventions group while the other Yes-levels are lower. This inconsistency across response levels between groups increases the uncertainty of the estimate, widens the confidence interval and gives a higher and thus not significant p value.

As for the overall result at 12 months for the questions on memory, the odds ratios are all in the same direction, but the confidence intervals are wide and either overlap or are near 1 (which indicates no difference). The findings on memory could of course be discussed to a greater extent. For example, there are theoretical aspects of what is measured as the question on recalling facts and names are related to more specific and different memory domains while the poor memory is more global but also have to be seen in the light of the social environment of the participant. However, in light of this being exploratory analyses without correction for multiple testing we refrained from discussing each significant result. And we feel that it would be hard to elaborate in the discussion while keeping it concise and stringent to the main findings. We have

however revised the discussion with this brief addition:

One memory outcome reached statistical significance in the primary analysis, this at 12 months. The estimated effects were in the same direction across all the memory outcomes at 12 months, albeit with wide confidence intervals.

You state in the statistical plan, that you planned to control for depression and mood in the sensitivity analyses, but this is not stated in supplementary Table S4, please explain why.

Table S4 shows the per-protocol analysis, this was intended to be adjusted in the same way as the primary analysis. This is stated in the statistical analysis plan (supplement 1) but there the per-protocol is, however, not referred to as a sensitivity analysis. I have removed "sensitivity" from the title of table S4 and in the manuscript when referring to the per-protocol analysis.

As for the sensitivity analysis mentioned in the statistical analysis plan regarding memory outcomes and controlling for depressive mood, it was to be a separate analysis. We thought this would be of interest as there is an ongoing discussion on whether subjective complaints of cognitive dysfunction from cancer patients could represent symptoms of depression. And therefore, we wanted to explore if an effect from the intervention on self-reported memory function was indirect and mediated by psychological wellbeing. As we found no substantiate effect from the intervention, we decided this analysis was redundant. We recognise now that it could have been more clearly stated in the statistical plan that the sensitivity analysis was to evaluate mediation of an effect. And therefore, not planned to be carried out if no substantial effect was found.

Discussion

Your conclusion is valid based on design and methodology and the secondary analysis and you discuss most of the relevant limitations such as not using valid instruments for the outcomes.
